# Abdominal ultrasonography of the healthy howler monkey (*Alouatta fusca*)

**DOI:** 10.1002/vms3.54

**Published:** 2017-01-23

**Authors:** Raquel Sartor, Thiago R. Müller, Maria J. Mamprim, Carlos R. Teixeira, Livia P. de Souza, Ricardo C. Lehmkuhl, Marilia G. Luciani

**Affiliations:** ^1^ School of Veterinary Medicine and Animal Science – FMVZ São Paulo State University – UNESP Rubião Júnior District s/n Botucatu São Paulo 18618‐970 Brazil; ^2^ Veterinary Diagnostic Imaging Section Agroveterinary Sciences Center of Santa Catarina State University Botucatu São Paulo Brazil; ^3^ Universidade Estadual do Centro‐oeste – UNICENTRO Guarapuava Paraná Brazil

**Keywords:** abdomen, diagnostic imaging, non‐human primate, normal, ultrasound

## Abstract

Abdominal ultrasonography was performed in six healthy adult brown howler monkeys *Alouatta fusca* and the normal ultrasonographic anatomy of the liver, gallbladder, stomach and urinary tract described for the first time. Findings were compared with post‐mortem studies. The renal cortex was isoechoic to the spleen and isoechoic or hyperechoic to the liver. Kidney length and renal arterial resistive index, systolic and diastolic velocity were calculated. The liver showed a homogeneous hypoechogenic echotexture. The aim of this study was to describe the normal abdominal viscera echoanatomy providing information of normal abdominal anatomical structures in the howler monkey.

## Introduction

More than 24 species of primates can be found in the Atlantic Forest of Brazil, a tropical dry forest extending along the coast of Brazil and inland to Paraguay and Argentina (Bicca *et al*. 2006). Howler monkeys Alouatta spp have a wide distribution within various biomes are found in the neotropical region. Due to extensive destruction of their natural habitat, they are considered threatened (Gregorin 2006; Mendes *et al*. 2003). Alouatta fusca is also known as Alouatta guariba and it was listed as critically endangered by the IUCN red list, but is it now classified as least concern (Mendes *et al*. 2008).

Anatomical studies and information on endangered species have been shown to be important in conservation efforts (Costa *et al*. 2005; Lindenmayer *et al*. 2000). Howler monkeys are free roaming wild animals in Brazil and South America, and they are frequently involved in road accidents and are sometimes presented to small animal or wildlife veterinarians with injuries. Howler monkeys are commonly kept in captivity and therefore knowledge of normal ultrasonographic anatomy is useful not only for diagnosis of disease in injured wild monkeys but also in captive populations.

One of the most commonly used and effective tools for studying the architecture of the abdominal organs is ultrasonography. Sonographic features of normal anatomy and lesions of organs are widely reported in domesticated species. Exotic species ultrasound is more often used for monitoring pregnancy, sex determination in monomorphic species, evaluation of abnormal structures and guided biopsies (Redrobe 2008).

Ultrasonography may prove to be a valuable diagnostic screening tool for multiple disease processes in the *Alouatta* genus. This study aims to describe the normal sonographic anatomy of the liver, stomach and urinary tract of the *Alouatta fusca*.

## Material and methods

### Humane care guidelines

The monkeys were kept in a safe, clean and calm enclosure. They were fed twice a day with fruits, leaves, vegetables and nuts. The study and ultrasound examination was performed during the annual checkup where monkeys were also tested for Mycobacterium tuberculosis.

### Sample population and immobilization

The howler monkeys were all originally free‐living and were rescued from urban areas near São Paulo and Paraná (Southern and Southeastern territory of Brazil). The males were kept separated from the females to avoid possible territory and dominance disputes. Six captive *Alouatta fusca* were submitted for abdominal ultrasonographic examination. They consisted of four non‐sexually active mature males, and two non‐gravid mature females from the Medical and Research Center of Wild Animals (CEMPAS), School of Veterinary Medicine and Animal Science of the São Paulo State University. The howler monkeys were scanned by a single ultrasonographer (R.S.) over a 2 day period in May 2012. All howler monkeys were fasted for 12 h prior to scanning. Access to water was not restricted. On the day of the scanning, weights were recorded. Health status was determined by long‐term history, clinical and laboratory examinations. Blood sampling was performed by femoral venipuncture. Complete blood count, kidney and liver function tests were performed.

All monkeys were sedated with a combination of ketamine hydrochloride (Ketamin, Cristália, Itapira, São Paulo, Brazil) (8 mg/kg) and midazolam hydrochloride (Dormire, Cristália, Itapira, São Paulo, Brazil) (0.4 mg/kg), administered intramuscularly (Carpenter, 2012), concurrent maintenance fluid therapy was provided by catheterization of the femoral vein (lactated Ringer's solution, 10 mL/kg/h).

### Ultrasonography

Immobilized howler monkeys were placed in dorsal recumbency, the abdomen was shaved, and sonographic coupling gel was applied. A triplex scan ultrasonographic (Logiq 3, General Electric Company, Milwaukee, Wisconsin, 53201, United States of America) device, with a multifrequency linear array probe of 6–10 MHz was used. The animals were examined in left lateral, right lateral and dorsal recumbencies. Ultrasound examination started with the evaluation of the urinary bladder, left kidney, spleen, liver, gallbladder, stomach and right kidney. Abdominal images of anatomical structures were evaluated for size, shape, position, echotexture and echogenicity. The bladder was visualized in transverse and longitudinal planes. Bladder wall thickness was measured dorsally. Kidney images were obtained in frontal planes, sagittal and transverse, to fully evaluate the anatomical position, length, shape, echotexture, echogenicity and relationship between the cortical and medullary region. The relationship between renal cortex and medulla was measured in the sagittal plane. Kidney length was obtained in frontal view. The liver was visualized in the sagittal and transverse planes with the transducer positioned at the ventral midline caudal to the xiphoid, directing it anteriorly. Images with the transducer between the last intercostal spaces were also obtained. Size, shape, echogenicity and echotexture of liver parenchyma beyond the wall and contents of the gallbladder were noted. The stomach was evaluated for gastric wall thickness and gastric contents were evaluated. Stomach wall thickness was measured between rugal folds. After B‐mode examination, colour Doppler and pulsed waved Doppler were used to evaluate renal vasculature.

A necropsy of a 6 kg adult male *Alouatta fusca* that died of head trauma was performed to compare ultrasonographic findings and location of abdominal organs. Ultrasound examination and histopathology of abdominal organs was not performed in this howler monkey. Association of sonographic images with anatomical specimens allowed identification of anatomical differences, better identification of the structures and ultrasound images corresponded with the anatomical specimens.

## Results

Mean ages of sampled male and female howler monkeys were not significant different (*P* < 0.001). All monkeys were between 5 and 6 years of age (mean female age = 5.3 years of age ± SD 0.2 years; mean male age = 5.5 years of age ± SD 0.7 years of age). Males had an average weight of 6 kg and average height of 39 cm long and females had an average weight of 4.5 kg and 38 cm long. All howler monkeys had a stress leukogram (monocytosis, lymphopenia and mild mature neutrophilia) which was attributed to their initial physical restraint and was presumed to be incidental. Bladder, kidneys, liver, gallbladder and stomach were visualized in all animals. Techniques for evaluation of abdominal organs and imaging findings are grouped and described individually below. Renal measurements are listed with arithmetic means and standard deviations in Table 1.

**Table 1:** Measurement of renal length and spectral doppler evaluation of left renal artery in *Alouatta fusca*

*Alouatta fusca*	RK (cm)	LK (cm)	*V*s (cm/sec)	*V*_d_ (cm/sec)	RI
1	3.76	3.84	28.12	12.89	0.54
2	4.26	4.11	30.73	13.84	0.55
3	4.56	4.14	34.30	16.00	0.53
4	3.85	3.3	29.54	12.99	0.56
5	3.50	3.80	31.33	13.98	0.46
6	3.12	3.48	31.16	13.85	0.56
Average (±SD)	3.84 (±0.52)	3.78 (±0.34)	30.86 (±2.07)	13.92 (±1.68)	0.53 (±0.03)

RK, length of the right kidney; LK, left kidney length; *V*_s_, peak systolic velocity; *V*_d_, end diastolic velocity; RI, resistivity index; SD, standard deviation.

### Urinary system

**Figure 1:**
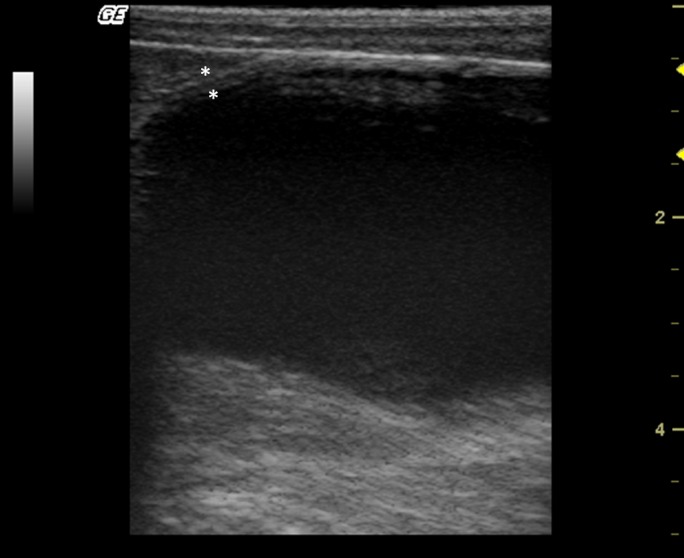
Longitudinal sonogram of a bladder in a female of *Alouatta fusca* presenting a homogeneous anechoic content, and a well‐defined echogenic thin wall.

The urinary bladder apex was observed as a circular or oval structure cranial to the pubis, with homogeneous anechoic content, and a well‐defined echogenic thin wall, average thickness of 0.18 cm (Fig. 1). In two males, suspended echogenic luminal debris was detected in the urinary bladder.

**Figure 2:**
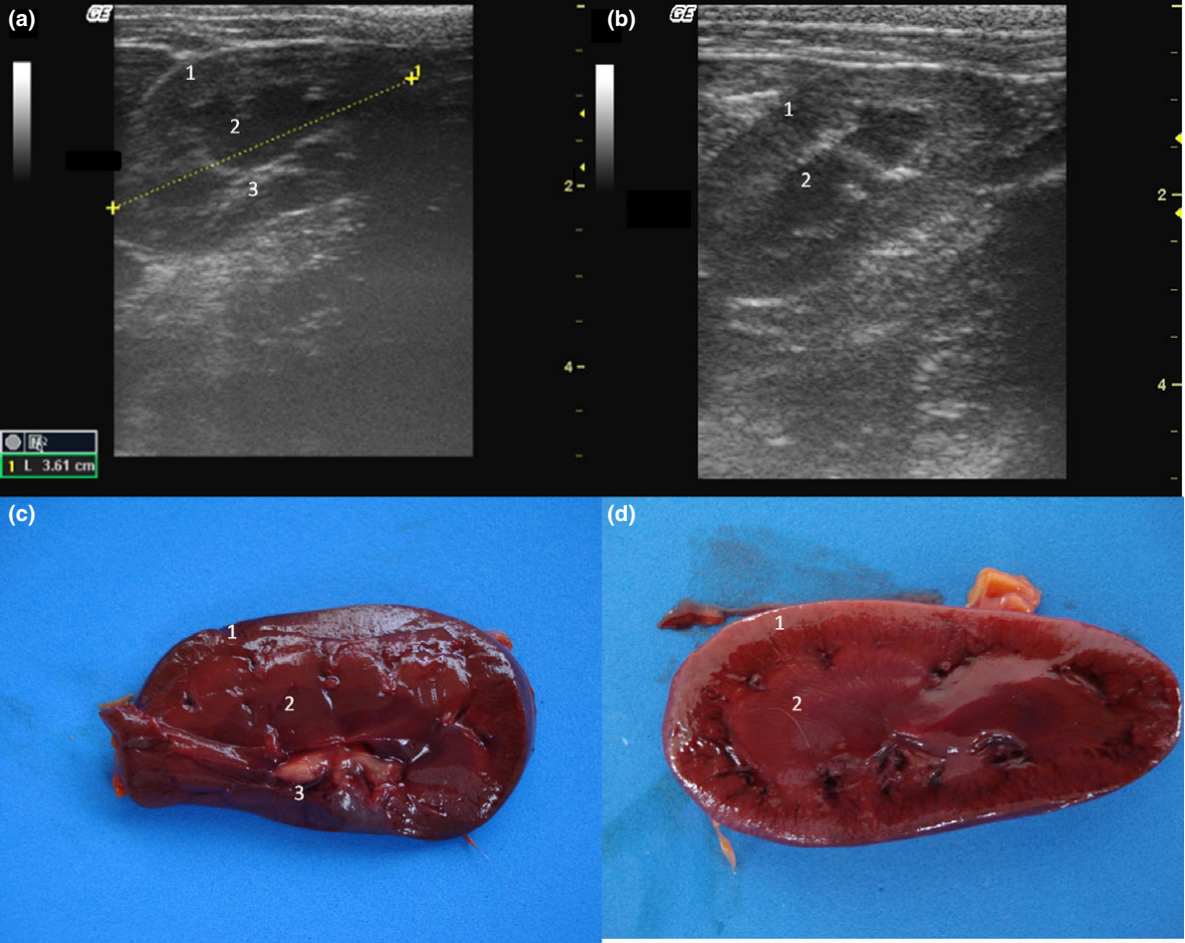
(a,b) Sonogram of the kidney in frontal (a) and sagittal (b) image planes. (c,d) Post‐mortem comparative study of the kidney on a frontal view (c) and sagittal view (d). 1 – Cortical, 2 – Medullar, 3 – Renal pelvis.

The kidneys were observed in the retroperitoneal space. The left kidney was evaluated caudal to the gastric fundus and the right was identified in the renal fossa of the right liver in all individuals. Kidneys lengths (average of 3.81 cm), are described in Table 1. The renal outlines were well defined and regular, delineated by a thin hyperechogenic line, the renal capsule (Fig. 2a,b). It was possible to identify and delineate the cortical, medullary and renal pelvis regions in all individuals. The cortex showed homogeneous echotexture and it was isoechoic to hyperechoic to the liver parenchyma and isoechoic when compared with the splenic parenchyma. The medullary region was well defined and appeared hypoechogenic in relation to the cortex. The pelvis was hyperechoic in relation to other parts of the kidney, possibly due to the fat present in this region. Discrete pyelectasis (0,2–3 mm) was observed in at least one kidney in five monkeys. This finding is assumed to be due to the fluid therapy or a normal variant. The relationship among cortical and medullary regions was measured, averaging proportion of 1:1. Post‐mortem study of the kidneys allowed a better understanding of the anatomy (Fig. 2c,d). Medullary rim sign was not observed in any individual.

**Figure 3:**
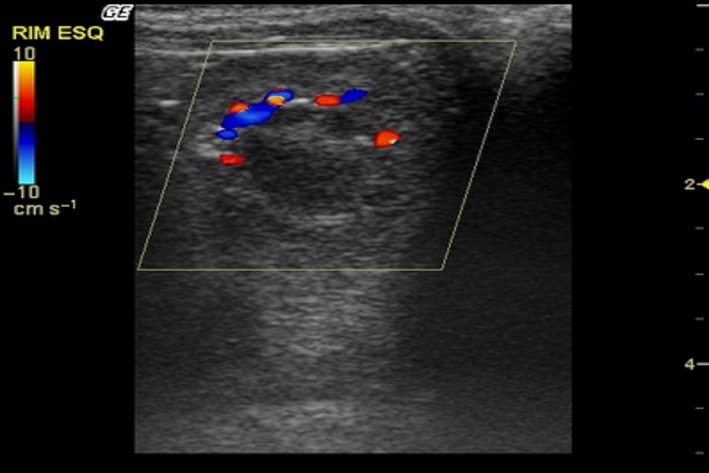
Color Doppler sonogram of the left kidney in an *Alouatta fusca*. Cortical and medullary regions were observed demonstrating arcuate vessels at the cortico‐medullary junction.

After the B‐mode examination, the kidneys were assessed by colour Doppler mode. The hilar and intrarenal vasculature was visualized. At the hilum, it was possible to define the renal artery and renal vein. Cortical and medullary regions were observed presenting several vascular branches, both arterial and venous, throughout the renal parenchyma (Fig. 3). No sign of turbulence was observed. Spectral Doppler mode examination measured systolic peak velocity (*V*_s_), end diastolic velocity (*V*_d_) and resistive index (RI [range: 46–56, mean 53]) of the renal artery. The spectral Doppler measurements of the left renal artery are described in Table 1.

### Liver and gallbladder

**Figure 4:**
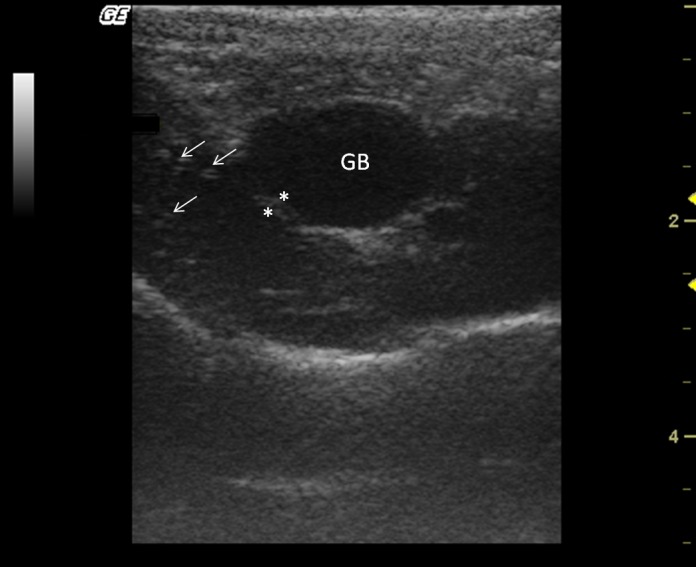
Transverse sonogram of the liver in an *Alouatta fusca*. The liver parenchyma presents a hypoechogenic ecotexture with hyperechogenic portal vessels walls, with the appearance of a “starry sky”. The gallbladder is filled with a homogeneous anechoic content, has a hyperechoic wall when compared with the hepatic parenchyma.

The liver size was assessed subjectively; it was located entirely within the rib cage, cranial to the stomach in all monkeys. All howler monkeys had liver parenchyma with homogeneous hypoechogenic echotexture compared with the cortex of the right kidney. Liver parenchyma also showed hyperechoic portal vessels walls (Fig. 4). The presence of gas in the stomach made it difficult to visualize the liver completely in two males.

**Figure 5:**
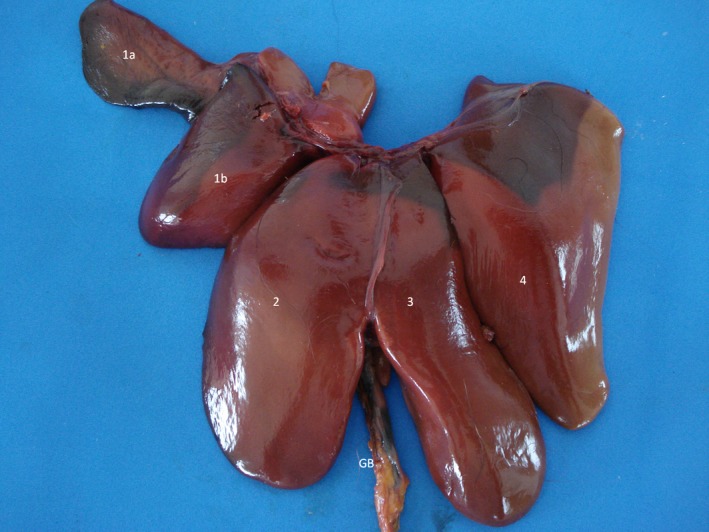
Post‐mortem preparation of *Alouatta fusca* liver (anterior view), showing the disposition of the gallbladder (GB) and hepatic lobes: (1a) papillary process of the caudate lobe, (1b) caudate process of the caudate lobe, (2) left lobe, (3) quadrate lobe, (4) right lobe.

The gallbladder had homogeneous anechoic fluid content; luminal debris was not detected. The gallbladder wall was totally or partially visualized as a hyperechoic line when compared with the hepatic parenchyma, average measuring 1.5 mm thick. Post‐mortem studies allowed identification of liver lobes: papillary process of the caudate lobe, caudate process of the caudate lobe, left lobe, quadrate lobe and right lobe; this allowed for a comparison of ultrasound imaging and anatomic position (Fig. 5).

### Stomach

**Figure 6:**
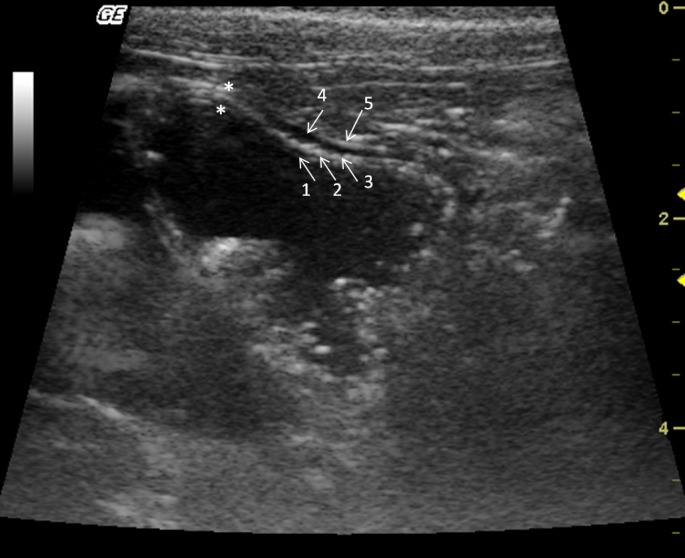
Transverse sonogram of the Alouatta fusca stomach. This structure is filled with anecoic (liquid) and hyperechogenic dots (food remains). The gastric wall has alternated echogenic pattern (arrows) (1) lumen; (2) mucosa; (3) submucosa; (4) muscularis; (5) serosa.

**Figure 7:**
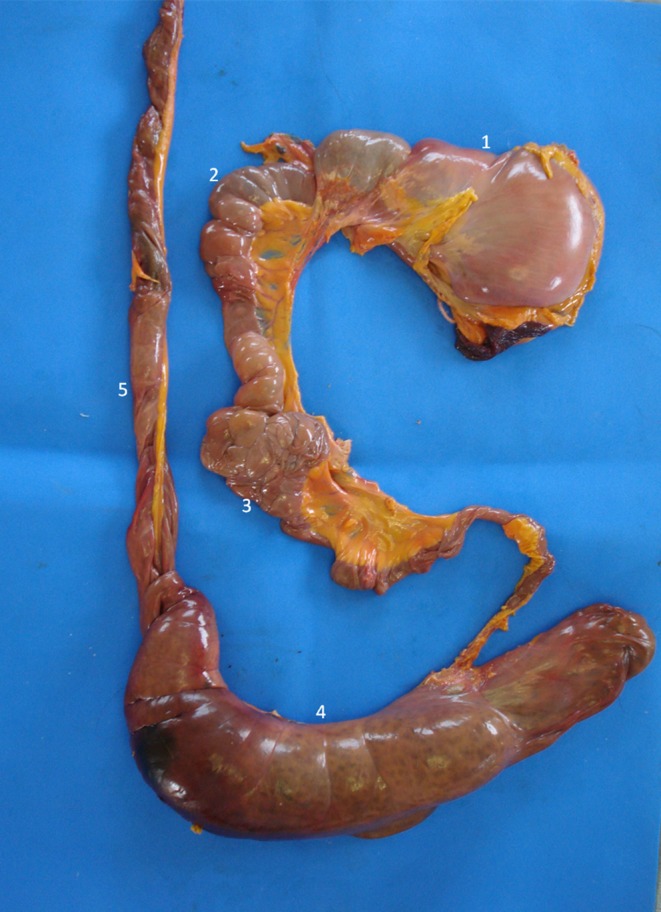
Post‐mortem study of the gastrointestinal tract of the *Alouatta fusca*. (1) Stomach, (2) Small bowel, (3) Pancreas, (4) Cecum, (5) Colon.

The stomach was observed caudal to the liver. Mild peristaltic activity was present, but it could not be quantitatively assessed because the animals were under anaesthesia. The stomach was distended by gas, and in spite of fasting, there was mild to moderate amount of anechoic content with hyperechogenic sediments, indicating the presence of water and food remnants. The average wall thickness was 2.8 mm. There were four layers alternating between hyperechogenic and hypoechogenic (Fig. 6). Stomach wall layering was detected in all individuals, the gastric mucosa and muscularis layers were anechoic, whereas the sub‐mucosa and serosa were hyperechoic. During post‐mortem examination the gastrointestinal tract structures were identified (Fig. 7).

## Discussion

The howler monkey is considered an endangered species in Brazilian territory mostly due to habitat destruction and illegal hunting (Bicca *et al*. 2006). Information regarding the abdominal sonographic anatomy of the howler monkey has not been reported. It is rare to find howler monkeys in research facilities (Costa *et al*. 2005; Gregorin 2006; Mendes *et al*. 2003). The small number of individuals that were submitted to sonographic examination is one limitation of this study. Another limitation was anaesthesia time, as the monkeys were sedated for their annual checkup; time was limited for obtaining ultrasound parameters of other organs. Further studies are needed in order for obtaining more parameters Figs 6, 7.

Non‐human primate species can often hide evident signs of illness from human caretakers and ultrasound examination may provide a useful diagnostic tool for detecting and identifying diseases in these species (Bailey & Mansfield 2010). Ultrasound at 10 MHz is a useful diagnostic modality to evaluate abdominal organs in howler monkeys.

The urinary bladder apex was identified in all howler monkeys cranially to the pubis and suspended echogenic debris detected in two males may be an incidental finding as it is considered to be in humans (Wachsberg *et al*. 1998). Clinical relevance of this finding cannot be assessed without additional analysis including urinalysis and urine culture.

Pyelectasis was observed in at least one kidney in five monkeys. This finding may be associated with diuresis, a full urinary bladder, fluid therapy, renal insufficiency, pyelonephritis and vesicoureteral reflux and/or outflow obstruction in human and veterinary patients (Bates 2004; Walsh & Dubbins 1996). Therefore, since the monkeys all had normal serum chemistry and complete blood count, the pyelectasis was attributed to fluid therapy and was believed to be clinically irrelevant.

The spectral Doppler evaluation of the renal vasculature was measured only in the left renal artery due to ease of access and anaesthesia time limitations as animals were immobilized for checkup. Obtaining B‐mode and Doppler measurements in both kidneys would have required an extension of the chemical restraint. The real effect of the anaesthesia on the Doppler values measured it is not known, so the measurements should be evaluated with caution considering the possible interference of the anaesthetic protocol used. These Doppler values are considered as complementary in renal ultrasonography. Values of resistivity index tend to be increased in parenchymal kidney diseases that progress to fibrosis, whereas smaller values tend to be related to acute inflammatory changes in small animals (Nyland & Mattoon 2002).

Sonographic hepatic parenchyma characteristics and location of the great vessels at the hepatic hilus are similar to normal findings in veterinary and human medicine (Bates 2004). The hyperechoic portal vessels walls and a prominent right liver is comparable to reports in New World primates such as common marmoset (*Callithrix jacchus*) (Wagner & Kirberger 2005) and humans (Bates 2004). Moreover, the gallbladder location between liver lobes in the right cranioventral abdomen and the lack of echogenic luminal debris, even with a 12‐h fast, is similar to the common marmoset's gallbladder (Wagner & Kirberger 2005).

The hypoechogenic hepatic parenchyma is also described in other species, such as dogs, and it may be associated with acute liver inflammatory processes, sepsis, cancer or anaesthetics administration (Nyland & Mattoon 2002) and it may be a normal finding (Ivančić & Mai 2008). Therefore, liver ultrasonographic appearance may be associated and it was attributed to anaesthesia as the monkeys were considered healthy and had normal serum chemistry and complete blood count as well as no history of previous illness.

Normal sonographic evaluation of the stomach has been described in the common marmoset (Wagner & Kirberger 2005) and primates are susceptible of gastrointestinal disease (Amory *et al*. 2013).

Currently there are no reports in the literature of the abdominal sonographic appearance *Alouatta* to compare and discuss the results. The description of the normal sonographic appearance of the organs in Alouatta and post‐mortem images are provided to supplement existing literature and for comparison in future cases.

Due to the specificity of the Alouatta genus and the great difficulty of working with wildlife, these results can be of practical and diagnostically useful in future research of renal, liver, gallbladder, stomach, and urinary bladder diseases. Ultrasound provides an excellent non‐invasive assessment of the howler monkey abdomen and may prove to be a valuable diagnostic screening tool for multiple diseases. These results could support diagnostic protocols and aid prognosis in howler monkeys. Hopefully this study will improve the quality in diagnostic imaging in captivity howler monkey and the maintenance and conservation of this endanger species. Further studies would be needed to prove the benefits of abdominal ultrasonography for diagnosis and staging of abdominal disease in howler monkeys.

## Source of funding

School of Veterinary Medicine and Animal Science of the São Paulo State University.

## Conflict of Interest

The authors declare that they have no conflicts of interest.

## Contributions

This work is the result of a contribution given by our former teacher and friend, Professor Olímpio Ribeiro Crisóstomo, a disciplined man dedicated to science who led a simple life, and has always honored Veterinary Medicine. May this be a late goodbye from a few of his friends.
